# Right Coronary Artery Thrombosis Causing ST-Elevation Myocardial Infarction After a Coronary Artery Disease Reporting and Data System Score of 0, With Disseminated Intravascular Coagulation During Idiopathic Acute Pancreatitis: A Case Report

**DOI:** 10.7759/cureus.116060

**Published:** 2026-09-10

**Authors:** Aoumar G Chamma, Wendy Saliba, Linda Chamma

**Affiliations:** 1 Cardiology, University of Balamand, Beirut, LBN; 2 Urology, Lebanese University, Beirut, LBN

**Keywords:** coronary ct angiography, disseminated intravascular coagulation, idiopathic acute pancreatitis, inferior st-elevation myocardial infarction, percutaneous coronary intervention, right coronary artery thrombosis

## Abstract

Acute pancreatitis may cause electrocardiographic abnormalities and troponin elevation that mimic acute myocardial infarction. True coronary thrombosis is less common. The development of disseminated intravascular coagulation shortly after percutaneous coronary intervention creates a difficult balance between bleeding control and prevention of early stent thrombosis.

A 68-year-old man with no previous pancreatitis presented with acute interstitial pancreatitis. Evaluation did not identify biliary disease, alcohol use, hypertriglyceridemia, hypercalcemia, medication exposure, autoimmune pancreatitis, pancreatic duct abnormalities, or malignancy, and the episode was classified as idiopathic. Coronary CT angiography performed five months earlier had shown an Agatston calcium score of 0 and Coronary Artery Disease Reporting and Data System (CAD-RADS) category 0, with no visible coronary plaque or stenosis. On day two of hospitalization, he developed inferior ST-elevation myocardial infarction caused by a 99% thrombotic subtotal occlusion of the mid-right coronary artery. Primary percutaneous coronary intervention with drug-eluting stent implantation restored normal coronary flow. Approximately 48 hours later, he developed bleeding-predominant disseminated intravascular coagulation with severe thrombocytopenia, hypofibrinogenemia, prolonged coagulation parameters, and mucocutaneous bleeding. Aspirin was continued, ticagrelor was changed to clopidogrel, and targeted blood-component replacement was administered. Acquired and inherited thrombophilia testing performed after recovery was negative.

Acute pancreatitis may mimic myocardial infarction, but true coronary thrombosis should be suspected when ischemic symptoms, reciprocal electrocardiographic changes, dynamic troponin elevation, regional wall-motion abnormality, and a culprit coronary lesion are present. A recent normal coronary CT angiogram does not exclude a later acute thrombotic event. Bleeding-predominant disseminated intravascular coagulation shortly after percutaneous coronary intervention requires individualized antiplatelet adjustment and targeted hemostatic support.

## Introduction

Acute pancreatitis is diagnosed when at least two of three features are present: characteristic abdominal pain, serum amylase or lipase elevation greater than three times the upper reference limit, and imaging findings consistent with pancreatic inflammation [1]. Most patients have interstitial edematous pancreatitis, but systemic inflammation may still cause cardiovascular, respiratory, renal, and hematologic complications [1,2].

Etiologic evaluation should address gallstones, alcohol consumption, hypertriglyceridemia, hypercalcemia, medication exposure, autoimmune disease, structural abnormalities, and malignancy [1]. When initial laboratory testing and imaging are unrevealing, further evaluation with magnetic resonance cholangiopancreatography or endoscopic ultrasound is recommended, particularly in older patients with a first episode of idiopathic acute pancreatitis [1].

Several indices and scales can help estimate early severity and guide monitoring. The Bedside Index for Severity in Acute Pancreatitis was originally described by Wu et al. and uses blood urea nitrogen, impaired mental status, systemic inflammatory response syndrome, age, and pleural effusion [3]. The systemic inflammatory response syndrome criteria were introduced by the American College of Chest Physicians/Society of Critical Care Medicine Consensus Conference [4]. Ranson criteria, Acute Physiology and Chronic Health Evaluation II, and the CT Severity Index were originally described by Ranson et al., Knaus et al., and Balthazar et al., respectively [5-7]. These scores add context, but repeated bedside reassessment remains essential.

Acute pancreatitis may be accompanied by various cardiac abnormalities, including electrocardiographic changes, troponin elevation, stress cardiomyopathy, coronary vasospasm, type 2 myocardial infarction, and pseudo-infarction patterns without coronary obstruction [8]. True acute coronary thrombosis has also been reported, but it is considerably less common [8].

A normal coronary CT angiogram is reassuring, especially when the Agatston calcium score is 0 and the study is classified as Coronary Artery Disease Reporting and Data System 0, but it does not completely eliminate future acute coronary risk [9,10]. This distinction is important when new ischemic symptoms develop after a recent normal scan.

Disseminated intravascular coagulation is characterized by systemic coagulation activation, dysregulated fibrinolysis, endothelial injury, and microvascular thrombosis, with progression to hemorrhage or organ dysfunction in overt disease. Acute pancreatitis may trigger this process through cytokine release, tissue-factor expression, platelet activation, endothelial injury, and impairment of physiologic anticoagulant pathways [11].

We describe idiopathic acute interstitial pancreatitis complicated by inferior ST-elevation myocardial infarction despite normal coronary CT angiography five months earlier, followed by bleeding-predominant overt disseminated intravascular coagulation shortly after coronary stent implantation.

## Case presentation

A 68-year-old man presented with severe epigastric pain radiating to the back for 10 hours. The pain was associated with nausea, repeated vomiting, anorexia, and mild dyspnea. He denied chest pain at presentation.

He had no previous episode of pancreatitis, recurrent unexplained abdominal pain, gallstone disease, pancreatic disorder, or family history of pancreatitis. His medical history included hypertension and dyslipidemia. He had no diabetes mellitus or known coronary artery disease. He was a former smoker with a 20-pack-year history and had stopped smoking eight years earlier. He denied regular alcohol intake or recent binge drinking.

Five months before admission, he had undergone coronary CT angiography for atypical chest discomfort. The examination showed an Agatston calcium score of 0, no visible coronary plaque or stenosis, and normal left main, left anterior descending, left circumflex, and right coronary arteries. The examination was classified as Coronary Artery Disease Reporting and Data System 0.

On admission, his temperature was 37.3°C, blood pressure was 138/82 mmHg, heart rate was 96 beats/minute, respiratory rate was 20 breaths/minute, and oxygen saturation was 97% on room air. Abdominal examination showed epigastric tenderness without guarding or rebound tenderness. Cardiac and pulmonary examinations were unremarkable.

Initial laboratory testing showed marked pancreatic enzyme elevation, leukocytosis, mild renal dysfunction, and elevated inflammatory markers (Table 1).

**Table 1:** Initial laboratory findings on admission IgG4: immunoglobulin G4.

Parameter	Patient value	Reference range	Interpretation
Lipase	2,860 U/L	13-60 U/L	Markedly elevated
Amylase	740 U/L	30-110 U/L	Markedly elevated
White blood cell count	16,800/µL	4,000-11,000/µL	Elevated
Hemoglobin	14.2 g/dL	13.5-17.5 g/dL	Normal
Platelet count	236,000/µL	150,000-450,000/µL	Normal
Creatinine	1.4 mg/dL	0.7-1.3 mg/dL	Mildly elevated
Blood urea nitrogen	26 mg/dL	7-20 mg/dL	Elevated
Fasting glucose	154 mg/dL	70-99 mg/dL	Elevated
Hemoglobin A1c	5.3%	<5.7%	Normal
Lactate dehydrogenase	346 U/L	140-280 U/L	Elevated
C-reactive protein	46 mg/L	<5 mg/L	Elevated
Triglycerides	138 mg/dL	<150 mg/dL	Normal
Corrected calcium	9.1 mg/dL	8.5-10.5 mg/dL	Normal
Alanine aminotransferase	52 U/L	7-56 U/L	Normal
Aspartate aminotransferase	61 U/L	10-40 U/L	Mildly elevated
Alkaline phosphatase	108 U/L	44-147 U/L	Normal
Gamma-glutamyl transferase	72 U/L	8-61 U/L	Mildly elevated
Total bilirubin	1.1 mg/dL	0.2-1.2 mg/dL	Normal
Serum ethanol	Negative	Negative	No ethanol detected
Serum IgG4	62 mg/dL	4-86 mg/dL	Normal

Fasting glucose was elevated at 154 mg/dL, but hemoglobin A1c was 5.3%, supporting stress hyperglycemia rather than previously undiagnosed diabetes. Triglyceride and corrected calcium levels were within the reference range. Liver tests showed only mild aspartate aminotransferase and gamma-glutamyl transferase elevation without hyperbilirubinemia. Serum ethanol was negative, and serum immunoglobulin G4 (IgG4) was within the reference range.

The admission electrocardiogram showed sinus rhythm without ischemic changes. High-sensitivity troponin I was 12 ng/L, below the institutional 99th percentile upper reference limit of 34 ng/L.

Contrast-enhanced abdominal CT showed acute interstitial edematous pancreatitis with mild peripancreatic fat stranding. There was no pancreatic necrosis, organized collection, vascular complication, or pancreatic mass (Figure 1).

**Figure 1:**
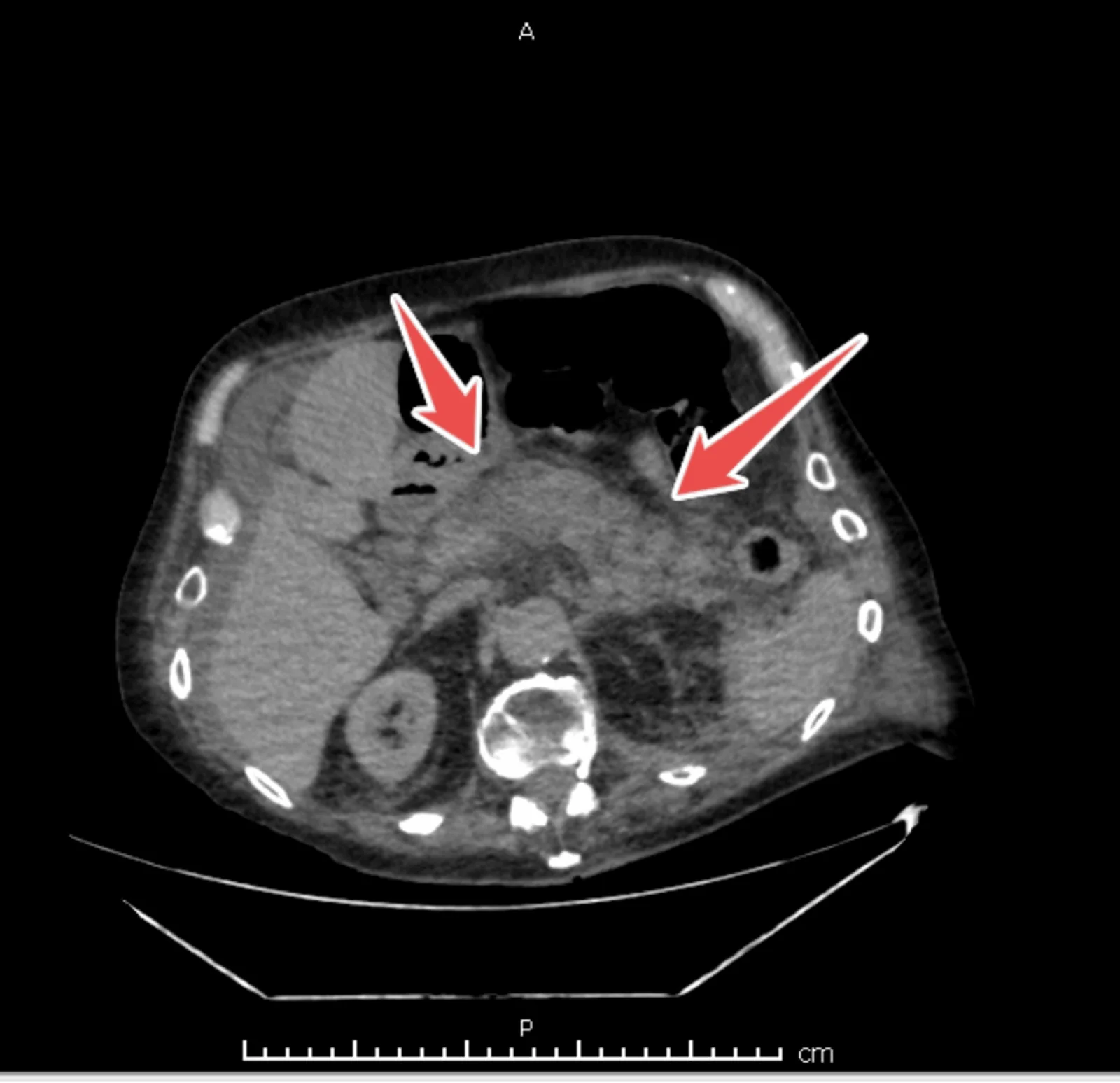
Axial contrast-enhanced CT demonstrates peripancreatic inflammatory fat stranding without pancreatic necrosis or organized peripancreatic collection. The inflammatory changes are highlighted by red arrows.

Abdominal ultrasound showed no gallstones, biliary sludge, gallbladder-wall thickening, or common bile duct dilatation. The common bile duct measured 5 mm. Magnetic resonance cholangiopancreatography showed no choledocholithiasis, biliary obstruction, pancreatic duct dilatation, pancreas divisum, or pancreatic lesion.

The coronary CT angiography performed five months earlier was reviewed again and confirmed normal coronary anatomy without visible plaque, calcification, or stenosis (Figure 2).

**Figure 2:**
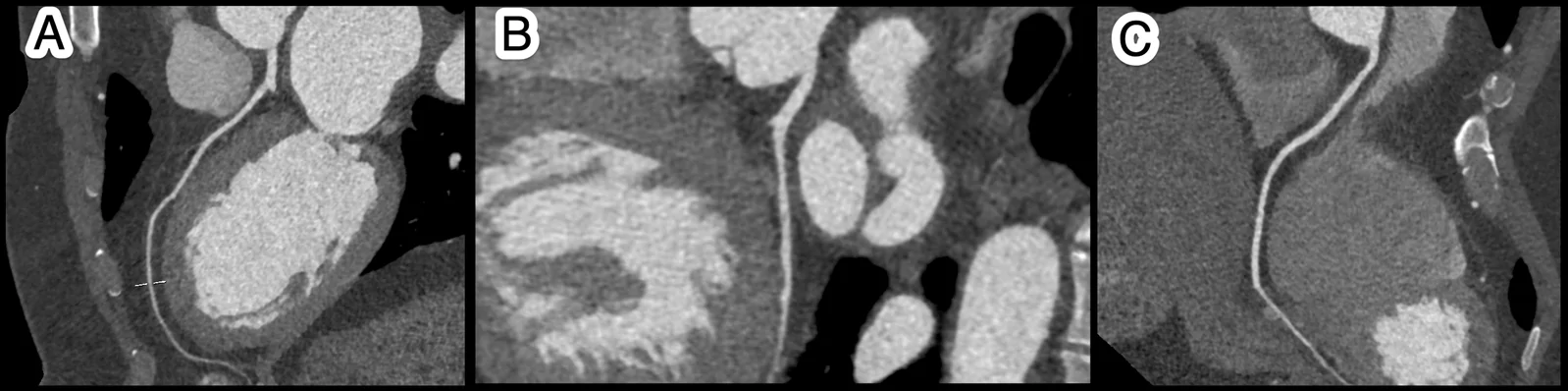
Normal coronary CT angiography performed five months before admission. A: Left anterior descending artery; B: left circumflex artery; C: right coronary artery. The Agatston calcium score was 0, and the examination was classified as Coronary Artery Disease Reporting and Data System 0.

Alcohol-related, biliary, hypertriglyceridemic, hypercalcemic, autoimmune, structural, malignant, and medication-related causes of pancreatitis were not identified. His long-term medications were amlodipine and atorvastatin, without recent changes. There was no recent exposure to thiazides, valproate, azathioprine, corticosteroids, glucagon-like peptide-1 receptor agonists, or new antibiotics.

The episode was classified as a first episode of idiopathic acute interstitial pancreatitis. Hereditary pancreatitis testing was not pursued because of his age, absence of recurrent attacks, and negative family history.

His Bedside Index for Severity in Acute Pancreatitis score was 3. He had no altered mental status and no pleural effusion on admission imaging. The score was based on age older than 60 years, blood urea nitrogen above 25 mg/dL, and systemic inflammatory response syndrome positivity from tachycardia and leukocytosis. Systemic inflammatory response syndrome criteria were met by a heart rate above 90 beats/minute and a white blood cell count above 12,000/µL. The Ranson score at 48 hours was two, based on age older than 55 years and admission white blood cell count above 16,000/µL. The Acute Physiology and Chronic Health Evaluation II score was 7, and the CT Severity Index was 2. There was no persistent organ failure or pancreatic necrosis.

He was treated with intravenous lactated Ringer’s solution, analgesia, antiemetics, and early oral refeeding as his symptoms improved. Antibiotics were not administered because there was no evidence of cholangitis, infected pancreatic necrosis, bacteremia, or another bacterial infection.

On hospital day two at approximately 08:00 hrs, his temperature increased to 38.4°C, white blood cell count to 19,600/µL, and C-reactive protein to 164 mg/L. Blood pressure remained stable, and lactate was 1.8 mmol/L. Blood cultures were obtained. The findings were attributed to pancreatitis-related systemic inflammation. Procalcitonin was not elevated at 0.01 ng/mL, with a reference range of less than 0.05 ng/mL. Urinalysis was unrevealing, chest imaging showed no pneumonia, and blood cultures remained negative.

At approximately 14:00 hrs on the same day, he developed sudden retrosternal chest pain radiating to the left arm, accompanied by diaphoresis and nausea. Blood pressure was 126/78 mmHg, heart rate was 112 beats/minute, and oxygen saturation was 95% on room air.

Electrocardiography showed 3 mm ST-segment elevation in leads II, III, and arteriovenous fistula (aVF), with reciprocal 1.5-2 mm ST-segment depression in augmented vector left (aVL). There was no significant ST-segment depression in V1-V3. Right-sided leads were recorded, and V4R showed no diagnostic ST-segment elevation (Figure 3).

**Figure 3:**
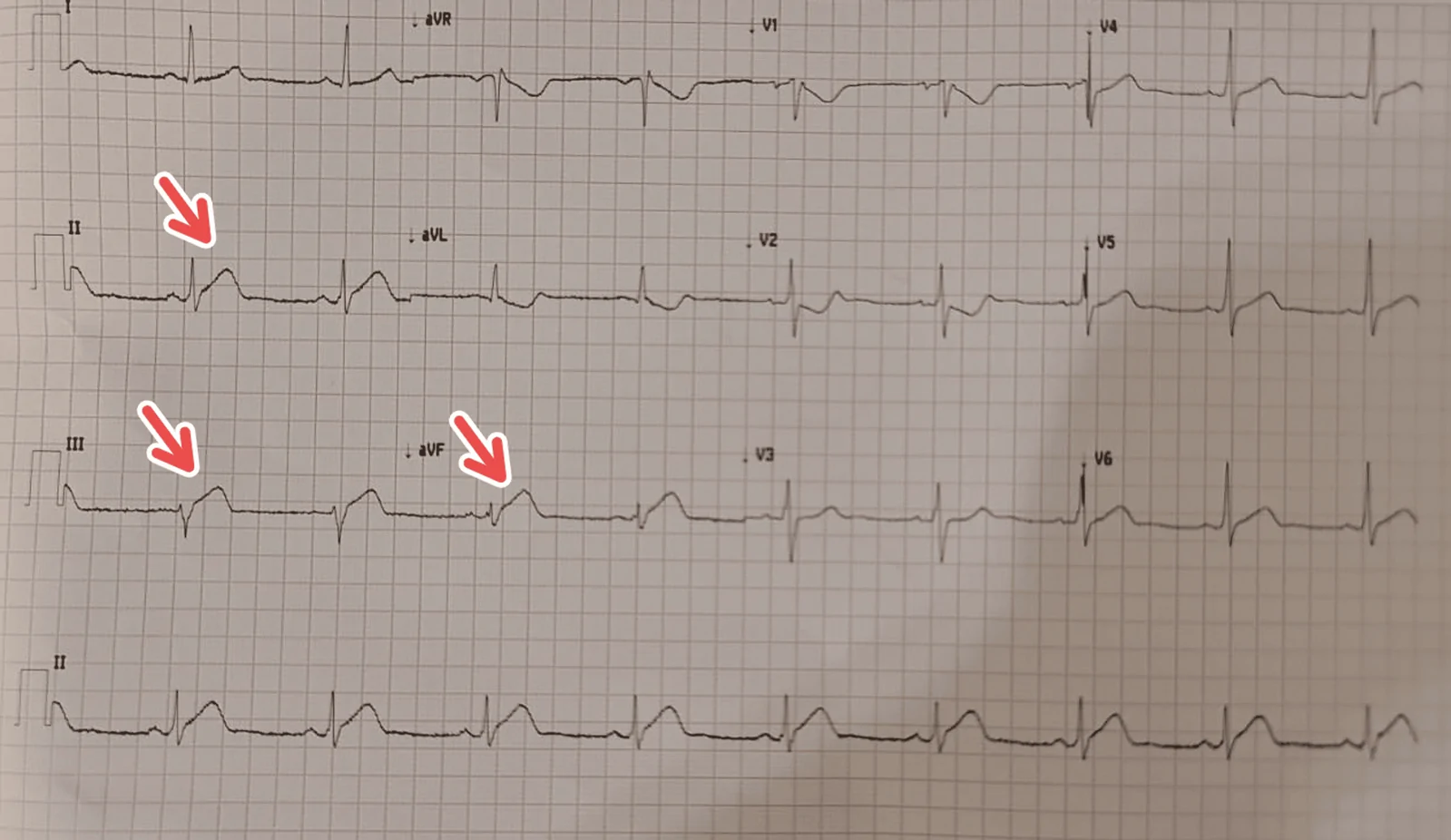
Electrocardiogram showing acute inferior ST-elevation myocardial infarction. Twelve-lead electrocardiogram obtained during acute chest pain demonstrates ST-segment elevation in leads II, III, and aVF, with reciprocal ST-segment depression in aVL. The inferior ST-segment elevations are highlighted by red arrows. aVF: arteriovenous fistula; aVL: augmented vector left.

High-sensitivity troponin I increased to 2,460 ng/L at chest-pain onset, 18,900 ng/L after six hours, and a peak of 42,700 ng/L after 14 hours. It decreased to 21,300 ng/L at 24 hours.

Bedside transthoracic echocardiography showed mildly reduced left ventricular systolic function, with an estimated ejection fraction of 48%, and basal and mid-inferior wall hypokinesia. The right ventricle was not dilated but showed mildly reduced systolic function. There was no significant valvular disease, intracardiac thrombus, pericardial effusion, or mechanical complication.

The patient was transferred directly to the catheterization laboratory. Electrocardiogram-to-balloon time was 54 minutes. Coronary angiography was performed through right radial access. The left main coronary artery was normal. The left anterior descending artery had mild luminal irregularities without obstructive stenosis. The non-dominant left circumflex artery had no significant disease.

The dominant right coronary artery showed a 99% thrombotic subtotal occlusion in the mid-segment, with Thrombolysis in Myocardial Infarction (TIMI) 1 distal flow. The angiographic thrombus burden was TIMI thrombus grade 3. There was no severe calcification or angiographic evidence of diffuse chronic coronary disease (Figure 4).

**Figure 4:**
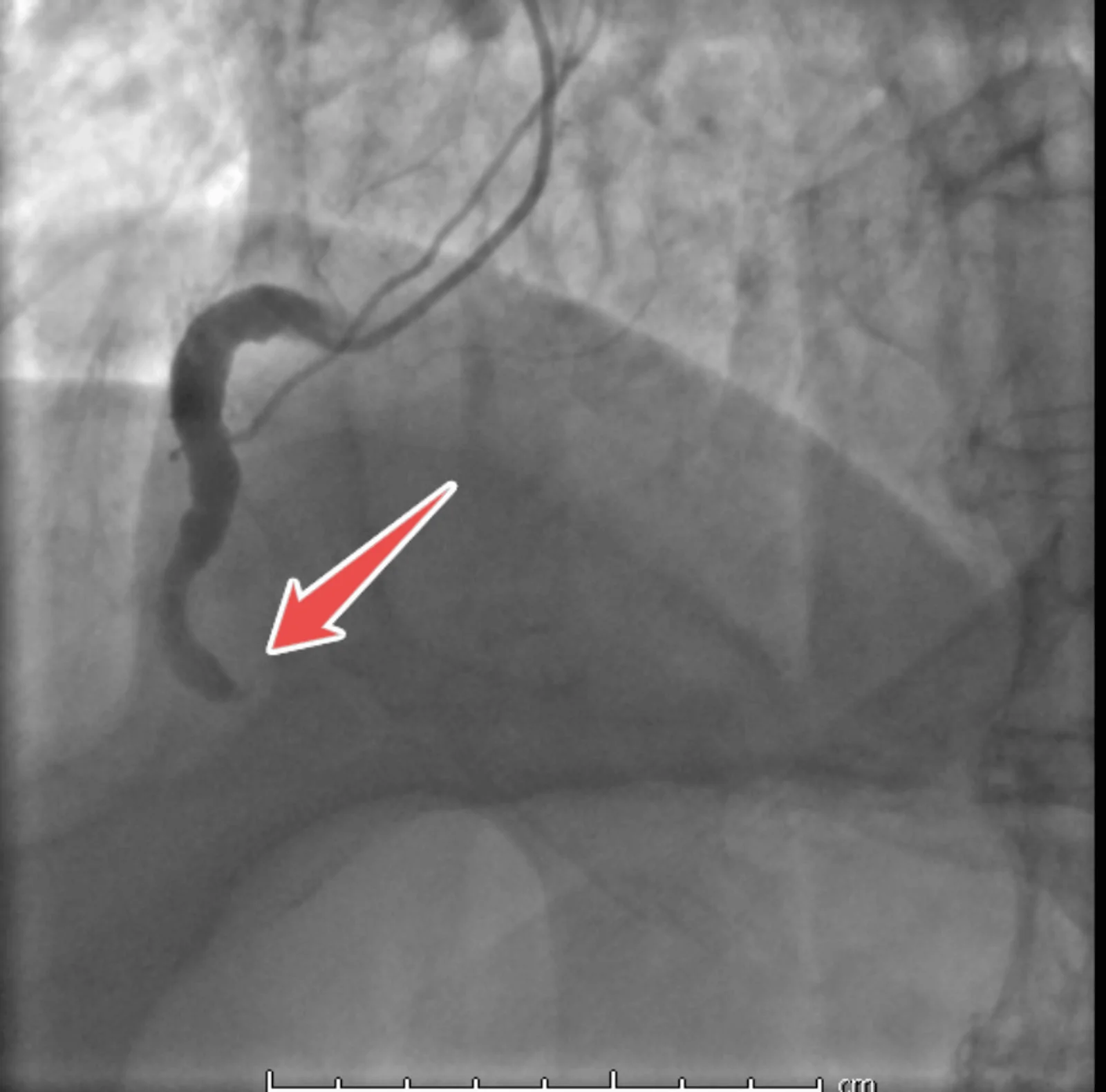
Coronary angiography demonstrates a 99% thrombotic subtotal occlusion of the mid-right coronary artery with TIMI 1 distal flow. The culprit lesion is highlighted by a red arrow. TIMI: Thrombolysis in Myocardial Infarction.

The patient received aspirin 300 mg, ticagrelor 180 mg, and unfractionated heparin 70 U/kg. A glycoprotein IIb/IIIa inhibitor was not administered because of active pancreatitis and concern for bleeding.

After guidewire passage, the lesion was predilated with a 2.5×15 mm semi-compliant balloon. A 3.5×24 mm everolimus-eluting drug-eluting stent was implanted in the mid-right coronary artery and post-dilated with a 3.75 mm non-compliant balloon.

Final angiography showed TIMI 3 flow, no significant residual stenosis, no edge dissection, and no no-reflow phenomenon (Figure 5).

**Figure 5:**
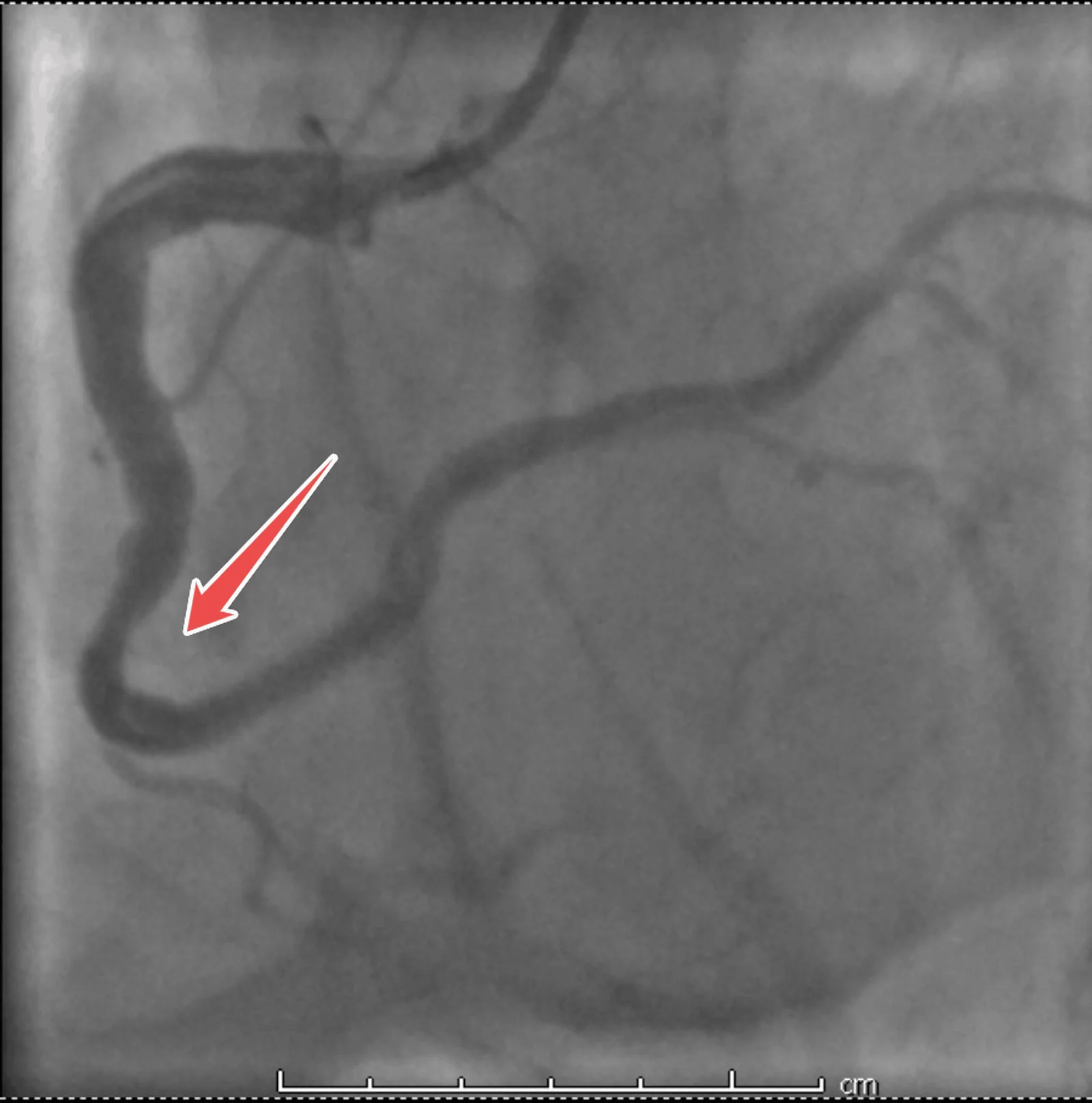
Final coronary angiography after percutaneous coronary intervention of the mid-right coronary artery. Final angiography after drug-eluting stent implantation shows restoration of TIMI 3 flow, no significant residual stenosis, no angiographic dissection, and no no-reflow phenomenon. The treated mid-right coronary artery segment is highlighted by a red arrow. TIMI: Thrombolysis in Myocardial Infarction.

Intravascular ultrasound and optical coherence tomography were not performed. The operator prioritized rapid reperfusion and avoided additional instrumentation in a thrombotic primary percutaneous coronary intervention lesion.

After percutaneous coronary intervention, he received aspirin 100 mg daily, ticagrelor 90 mg twice daily, atorvastatin 80 mg daily, and bisoprolol as tolerated. Heparin was discontinued after the procedure and was not reversed because there was no active bleeding.

On hospital day four at approximately 15:00 hrs, close to 48 hours after percutaneous coronary intervention, he developed diffuse ecchymoses over the upper limbs, venipuncture-site oozing, gingival bleeding, and pallor. There was no hematemesis, melena, hematuria, intracranial bleeding, or radial access-site hematoma.

Hemoglobin decreased from 13.8 g/dL to 10.7 g/dL. Platelet count fell from 218×10³/µL to 46×10³/µL. Prothrombin time was 25.8 seconds, compared with a laboratory control of 12.0 seconds, giving a prolongation of 13.8 seconds. International normalized ratio was 2.3, activated partial thromboplastin time was 58 seconds, and fibrinogen was 82 mg/dL. D-dimer was greater than 20,000 ng/mL fibrinogen-equivalent units, with a laboratory upper reference limit of 500 ng/mL fibrinogen-equivalent units. Fibrin degradation products were markedly elevated, and the peripheral blood smear showed occasional schistocytes. The laboratory results are summarized in Table 2.

**Table 2:** Evolution of hematologic and coagulation parameters before, during, and after disseminated intravascular coagulation. aPTT: activated partial thromboplastin time; DIC: disseminated intravascular coagulation; FEU: fibrinogen-equivalent units; INR: international normalized ratio; PCI: percutaneous coronary intervention; PT: prothrombin time; FEU: fibrinogen-equivalent units.

Parameter	Reference range	Admission	Post-PCI	DIC onset, Day 4	Day 6	Day 8
Hemoglobin	13.5-17.5 g/dL	14.2 g/dL	13.8 g/dL	10.7 g/dL	10.9 g/dL	11.4 g/dL
Platelet count	150-450×10³/µL	236×10³/µL	218×10³/µL	46×10³/µL	92×10³/µL	154×10³/µL
PT	11.0-13.5 seconds	12.2 seconds	12.6 seconds	25.8 seconds	16.9 seconds	12.8 seconds
INR	0.8-1.2	1	1	2.3	1.4	1.1
aPTT	25-35 seconds	29 seconds	31 seconds	58 seconds	39 seconds	31 seconds
Fibrinogen	200-400 mg/dL	348 mg/dL	322 mg/dL	82 mg/dL	168 mg/dL	286 mg/dL
D-dimer	<500 ng/mL FEU	420 ng/mL FEU	680 ng/mL FEU	>20,000 ng/mL FEU	8,900 ng/mL FEU	2,700 ng/mL FEU

Using the 2025 International Society on Thrombosis and Haemostasis updated disseminated intravascular coagulation scoring framework, the score was 8. Platelet count below 50×10⁹/L contributed two points, D-dimer greater than seven times the upper reference limit contributed three points, prothrombin time prolongation of at least six seconds contributed two points, and fibrinogen below 100 mg/dL contributed one point. The clinical phenotype was bleeding-predominant.

Pancreatitis-related systemic inflammation was considered the most likely trigger. Blood cultures remained negative, and there was no evidence of shock, contrast reaction, transfusion reaction, or procedure-related major hemorrhage.

Heparin-induced thrombocytopenia was considered. His 4Ts score was 2. A platelet reduction greater than 50% with a nadir above 20,000/µL contributed two points. Onset within the first four days without recent heparin exposure, absence of new thrombosis, and the presence of overt disseminated intravascular coagulation as a definite alternative explanation each contributed 0 points. Heparin-induced thrombocytopenia testing was not pursued because the pretest probability was low.

Cardiology, hematology, gastroenterology, and critical care teams jointly reviewed the case. As the coronary stent had been implanted only 48 hours earlier and bleeding was mucocutaneous without hemodynamic instability or critical-organ hemorrhage, complete interruption of dual antiplatelet therapy was avoided.

Aspirin 100 mg daily was continued. Ticagrelor was discontinued and replaced with clopidogrel 75 mg daily without a loading dose because of active bleeding. Systemic anticoagulation was not started because there was no venous thromboembolism, mechanical circulatory support, or thrombotic-predominant disseminated intravascular coagulation.

The patient received 10 units of cryoprecipitate, two units of fresh frozen plasma, and one adult dose of pooled platelets. Packed red blood cell transfusion was not required. Tranexamic acid was avoided because of the recent ST-elevation myocardial infarction and newly implanted coronary stent.

Bleeding stopped during the following 72 hours. Platelet count increased to 92×10³/µL on day 6 and 154×10³/µL on day 8. Fibrinogen increased to 168 mg/dL after cryoprecipitate and reached 286 mg/dL by day 8. International normalized ratio decreased to 1.4 on day 6 and 1.1 on day 8. D-dimer declined but remained above the reference range at discharge.

Repeat echocardiography before discharge showed improvement in inferior wall motion and an estimated left ventricular ejection fraction of 52%, without mechanical complications.

He was discharged on hospital day 10 on aspirin 100 mg daily, clopidogrel 75 mg daily, atorvastatin 80 mg daily, bisoprolol 2.5 mg daily, ramipril 1.25 mg daily, and proton-pump inhibitor therapy. Creatinine improved to 1.1 mg/dL before discharge, and renal function and potassium monitoring were arranged after ramipril initiation.

Outpatient endoscopic ultrasound performed six weeks later showed no microlithiasis, common bile duct stone, pancreatic mass, chronic pancreatitis, or structural pancreaticobiliary abnormality. The pancreatitis remained classified as idiopathic.

After complete resolution of the disseminated intravascular coagulation, a hypercoagulability evaluation was performed at 12 weeks. Lupus anticoagulant testing by dilute Russell viper venom time and silica clotting time was negative. Anticardiolipin IgG and IgM and anti-β2-glycoprotein I IgG and IgM antibodies were negative. Factor V Leiden and prothrombin G20210A mutations were absent. Protein C activity, free protein S antigen, and antithrombin activity were normal. Fasting homocysteine was normal. Janus kinase 2 V617F testing was negative, and flow cytometry showed no paroxysmal nocturnal hemoglobinuria clone. No acquired or inherited thrombophilia was identified. The clinical sequence is summarized in Table 3.

**Table 3:** Clinical timeline ACE: angiotensin-converting enzyme; DIC: disseminated intravascular coagulation; ECG: electrocardiogram; INR: international normalized ratio; PCI: percutaneous coronary intervention; STEMI: ST-elevation myocardial infarction; TIMI: thrombolysis in myocardial infarction.

Time point	Clinical event
Day 1	The patient was admitted with idiopathic acute interstitial pancreatitis. Initial ECG showed no ischemic changes, and high-sensitivity troponin I was within the institutional reference range.
Day 2, 08:00	Fever and rising inflammatory markers developed without a confirmed infectious source. Blood cultures were obtained and later remained negative.
Day 2, 14:00	The patient developed acute chest pain with inferior ST-segment elevation and reciprocal changes, consistent with inferior STEMI.
Day 2, afternoon	Coronary angiography showed thrombotic subtotal occlusion of the mid-right coronary artery. Primary PCI with drug-eluting stent implantation restored TIMI 3 flow.
Day 4, 15:00	Bleeding-predominant overt DIC developed approximately 48 hours after PCI, with thrombocytopenia, hypofibrinogenemia, prolonged coagulation parameters, and mucocutaneous bleeding.
Days 4-5	Aspirin was continued, ticagrelor was changed to clopidogrel, and the patient received cryoprecipitate, fresh frozen plasma, and platelet transfusion.
Day 6	Bleeding stopped, and platelet count, fibrinogen, and INR began to improve.
Day 8	Coagulation parameters continued to recover, with platelet count and fibrinogen approaching normal values.
Day 10	The patient was discharged in stable condition on dual antiplatelet therapy, high-intensity statin therapy, beta-blocker, low-dose ACE inhibitor, and proton-pump inhibitor therapy.
Week 6	Outpatient endoscopic ultrasound showed no occult microlithiasis, pancreatic mass, chronic pancreatitis, or structural pancreaticobiliary abnormality.
Week 12	Acquired and inherited thrombophilia testing was negative.

## Discussion

This case combines three uncommon events: idiopathic acute pancreatitis, thrombotic inferior ST-elevation myocardial infarction despite a recently normal coronary CT angiogram, and bleeding-predominant disseminated intravascular coagulation shortly after percutaneous coronary intervention. The temporal sequence suggests a possible relationship between systemic inflammation, coronary thrombosis, and coagulation failure.

The pancreatic episode remained idiopathic after abdominal CT, ultrasound, magnetic resonance cholangiopancreatography, IgG4 testing, and follow-up endoscopic ultrasound failed to identify a cause. Current guidance supports further imaging after a first idiopathic episode, particularly in patients older than 40 years, because occult microlithiasis and small pancreatic tumors may be missed during initial assessment [1]. Genetic pancreatitis testing was not pursued because the patient was 68 years old, had no previous episodes, and had no relevant family history.

The Bedside Index for Severity in Acute Pancreatitis score was three because the patient met systemic inflammatory response syndrome criteria through tachycardia and leukocytosis, in addition to age above 60 years and elevated blood urea nitrogen [3,4]. He had no altered mental status and no pleural effusion on admission imaging. The Ranson score was two, supported by age older than 55 years and admission white blood cell count above 16,000/µL [5]. Although imaging showed interstitial pancreatitis without necrosis, the elevated Bedside Index for Severity in Acute Pancreatitis score identified an increased early risk of deterioration.

The literature more commonly describes pancreatitis producing a pseudo-infarction pattern than true coronary occlusion. Reported pancreatitis-associated electrocardiographic abnormalities frequently involve inferior ST-segment elevation without obstructive coronary disease, with proposed mechanisms including electrolyte abnormalities, coronary vasospasm, stress cardiomyopathy, and inflammatory myocardial injury [8]. In the present case, typical ischemic pain, inferior ST-segment elevation with reciprocal changes, dynamic troponin elevation, inferior wall-motion abnormality, and a thrombotic subtotal occlusion of the dominant right coronary artery supported true myocardial infarction rather than a pancreatitis-related mimic.

Type 2 myocardial infarction was considered because the patient had fever, tachycardia, leukocytosis, and systemic inflammation. Type 2 myocardial infarction results from an imbalance between myocardial oxygen supply and demand without acute coronary atherothrombosis [12]. The discrete thrombotic right coronary artery lesion, TIMI 1 distal flow, TIMI thrombus grade 3 burden, and restoration of TIMI 3 flow after percutaneous coronary intervention favored type 1 myocardial infarction rather than isolated supply-demand mismatch [12-14].

A calcium score of 0 and Coronary Artery Disease Reporting and Data System 0 are associated with a very low probability of obstructive coronary disease at the time of coronary CT angiography, but they do not guarantee protection from a later acute event [9,10]. Small noncalcified lesions may be below the spatial resolution of CT, plaque biology may change, and systemic inflammation may increase endothelial activation and thrombogenicity. Because intravascular ultrasound and optical coherence tomography were not performed, the precise substrate, including plaque rupture, plaque erosion, or in-situ thrombosis without major plaque disruption, could not be determined.

The negative thrombophilia evaluation reduced the likelihood of antiphospholipid syndrome, common inherited thrombophilias, myeloproliferative neoplasm, and paroxysmal nocturnal hemoglobinuria. Testing was performed after the acute event and after normalization of coagulation parameters to reduce interference from disseminated intravascular coagulation and acute-phase changes. Even with negative testing, a transient inflammation-mediated hypercoagulable state remains biologically plausible. Acute pancreatitis can enhance thrombin generation, tissue-factor expression, platelet activation, and endothelial dysfunction [11].

The disseminated intravascular coagulation presentation was clinically important because it occurred after coronary stent implantation. Pancreatitis-associated coagulopathy can range from mild laboratory abnormalities to overt disseminated intravascular coagulation [11]. The 2025 International Society on Thrombosis and Haemostasis updated scoring framework was applied using its revised thresholds, and the score was eight [15]. The original International Society on Thrombosis and Haemostasis overt disseminated intravascular coagulation score was described by Taylor et al. [16]. In this patient, the marked reduction in fibrinogen, thrombocytopenia, ecchymoses, gingival bleeding, and venipuncture-site oozing supported a bleeding-predominant phenotype.

Heparin-induced thrombocytopenia was also considered because heparin had been used during percutaneous coronary intervention. The 4Ts score, described by Lo et al., was two, indicating low pretest probability [17]. Management was complicated by the recently implanted coronary stent. Contemporary acute coronary syndrome guidance recommends antiplatelet therapy after ST-elevation myocardial infarction treated with percutaneous coronary intervention, but bleeding risk may require individualized modification [18]. Complete withdrawal of antiplatelet therapy during the first days after stent implantation could have exposed the patient to acute stent thrombosis. Because bleeding was mucocutaneous and not life-threatening, aspirin was continued and ticagrelor was replaced with clopidogrel rather than stopping P2Y12 inhibition.

Randomized de-escalation trials provide only indirect support for switching from a potent P2Y12 inhibitor to clopidogrel. The Ticagrelor Versus Clopidogrel in Stabilized Patients With Acute Myocardial Infarction (TALOS-AMI) trial supported unguided ticagrelor-to-clopidogrel de-escalation after one month in stabilized acute myocardial infarction patients after percutaneous coronary intervention [19]. It did not study de-escalation 48 hours after percutaneous coronary intervention (PCI) during active disseminated intravascular coagulation. The decision in this case was therefore individualized based on bleeding severity and the immediate risk of stent thrombosis.

Systemic anticoagulation was avoided because the patient had bleeding-predominant disseminated intravascular coagulation without venous thromboembolism or another indication for anticoagulation. Tranexamic acid was avoided because of concern for coronary and stent thrombosis in the setting of a very recent ST-elevation myocardial infarction.

## Conclusions

Acute pancreatitis can produce electrocardiographic and biochemical abnormalities that mimic myocardial infarction, but true acute coronary thrombosis must remain in the differential diagnosis. In patients who develop ischemic chest pain associated with ST-segment elevation, reciprocal electrocardiographic changes, dynamic troponin elevation, and regional wall-motion abnormalities, urgent invasive coronary angiography should be pursued even when recent coronary CT angiography has shown no significant obstructive disease. In this case, angiography confirmed a thrombotic mid-right coronary artery occlusion requiring primary percutaneous coronary intervention. The subsequent development of bleeding-predominant disseminated intravascular coagulation shortly after stent implantation created a particularly challenging balance between hemorrhagic risk and prevention of early stent thrombosis. Management required individualized adjustment of antiplatelet therapy together with targeted replacement of coagulation components and close multidisciplinary monitoring. This case highlights the importance of promptly recognizing true coronary thrombosis during acute pancreatitis and carefully individualizing antithrombotic treatment when severe coagulopathy develops shortly after PCI.
